# Mechanism of the Micellar Solubilization of Curcumin by Mixed Surfactants of SDS and Brij35 via NMR Spectroscopy

**DOI:** 10.3390/molecules27155032

**Published:** 2022-08-08

**Authors:** Xiao Zhan, Zhaoxia Wu, Zhong Chen, Xiaohong Cui

**Affiliations:** Fujian Provincial Key Laboratory of Plasma and Magnetic Resonance, State Key Laboratory of Physical Chemistry of Solid Surfaces, Department of Electronic Science, Xiamen University, Xiamen 361005, China

**Keywords:** NMR spectroscopy, mixed micellar solubilization, curcumin, mechanism of micellar solubilization, relationship between structure and solubility

## Abstract

The micellar solubilization mechanism of curcumin by mixed surfactants of SDS and Brij35 was investigated at the molecular scale by NMR spectroscopy. Through the investigation of the micelle formation process, types and structures of mixed micelles and solubilization sites, the intrinsic factors influencing the solubilization capacity were revealed. For systems with α_SDS_ = 0.5 and 0.2, the obtained molar solubilization ratios (MSRs) are consistent with the MSR_ideal_ values. However, for α_SDS_ = 0.8, the solubilization capacity of curcumin is weakened compared to the MSR_ideal_. Furthermore, only one single mixed SDS/Brij35 micelles are formed for α_SDS_ = 0.5 and 0.2. However, for α_SDS_ = 0.8, there are separate SDS-rich and Brij35-rich mixed micelles formed. In addition, NOESY spectra show that the interaction patterns of SDS and Brij35 in mixed micelles are similar for three systems, as are the solubilization sites of curcumin. Therefore, for α_SDS_ = 0.5 and 0.2 with single mixed micelles formed, the solubility of curcumin depends only on the mixed micelle composition, which is almost equal to the surfactant molar ratio. Although curcumin is solubilized in both separate micelles at α_SDS_ = 0.8, a less stable micelle structure may be responsible for the low solubility. This study provides new insights into the investigation and application of mixed micelle solubilization.

## 1. Introduction

With the in-depth development of drug research, there is a gradually increasing trend in the number of new drugs with more complex molecular structures and consequently poorer solubility [1,2]. According to reports, as high as 90% of the drugs under research are water insoluble. The low solubility leads to poor bioavailability and absorption rates, which further decreases biological activity and greatly limits its application. Therefore, the development of feasible drug encapsulation schemes to improve the solubility of insoluble drugs is essential to achieving effective treatment. Until now, a variety of delivery systems have been developed, including solid dispersions [3], micelles [4], lipids [5], supramolecular assemblies [6], polymeric micelles [7,8], polyelectrolyte multilayer systems [9] and nano pharmaceutics [10,11]. Among them, micelles, which are formed by the self-assembling of surfactants at a concentration above a certain concentration (critical micellar concentration, CMC), are homogeneous and thermodynamically stable in water [12]. Due to the unique amphiphilic structure with a hydrophilic shell and a hydrophobic core, micelles are considered to be a simple and suitable medium for solubilizing insoluble drugs [13].

In addition to possessing the dual properties of two surfactants, mixed surfactant micelles may have completely different properties from those that the single surfactant itself has, such as a much lower CMC, thereby reducing the surfactant consumption, which is often beneficial in practical applications [14,15,16,17,18,19]. Besides, Mhenga et al. [20] found that there is a synergism effect in anionic/nonionic and cationic/nonionic binary surfactant mixtures. The solubility of hydrophobic drugs was significantly improved in mixed micelles [21,22]. One possible reason for the improved solubility is that mixed micelles are more likely to form a denser hydrophobic palisade layer than pure micelles, which allows hydrophobic drugs to localize more deeply [4]. In addition, it has been reported that insoluble drugs can be hosted by mixed micelles composed of glycerides, free fatty acids, bile salts, phospholipids and cholesterol [23,24]. Considering the effect of the mixed micellar structure on drug delivery, Mulrooney et al. [25] evaluated the impact of fatty acid (FA) type and phospholipid concentration on the formation and stability of mixed micelles. These indicate that not only in the delivery but also in the digestion of insoluble drugs, the role of the mixed surfactant micelles is crucial in affecting the bioavailability of poorly soluble drugs [26]. 

Although many studies have reported the synergistic effect of mixed micelles [4,27,28], mixed micellization may have an adverse effect on the solubilization of poorly water-soluble drugs [29]. To develop alternative and superior solubilization systems, various mixed micelle systems composed of different surfactants were investigated, and some were found to be inferior to pure micelles or classic mixed formulations [30]. Vinarov et al. reported that the mixed bile salt + ionic surfactant micelles cause a decrease in solubility [31]. These results suggest that mixed micellar systems may exhibit different solubilization behaviors due to the complicated interaction between different types of surfactants. Therefore, a better understanding of the molecular interaction between the surfactant–surfactant and surfactant–solute in mixed micellar solubilization systems, as well as the relationship between the solubility and interaction behaviors, is fundamental to the development of the solubilization formulations.

The solubility improvement of curcumin by surfactant micelles has been extensively investigated. Curcumin is an insoluble natural drug with biological and pharmacological activities such as antioxidant, antitumor and anticancer, and possesses broad medical application prospects in the treatment of various diseases. However, the low solubility and limited stability of curcumin result in its low bioavailability and absorption rate. To solve this problem, many solubilization schemes including single or mixed surfactant micelles for curcumin have been proposed [32,33,34,35,36]. Lapenna et al. [37] evaluated the solubilization of the low soluble drugs artemisinin and curcumin by the anionic surfactant sodium dodecyl sulfate (SDS) using ^1^H NMR and 2D diffusion ordering spectroscopy (DOSY) methods. Wang et al. [32] investigated the effects of the length and unsaturation of nonionic surfactants of Tween 20, Tween 60 and Tween 80 on the solubilization of curcumin. In addition, mixed micellar formulations composed of cationic and nonionic surfactants were investigated using fluorescence and conductivity techniques, showing a significantly enhanced solubility of curcumin [38]. 

In this study, with curcumin as a model drug, anionic surfactant SDS and nonionic polyoxyethylene 23 lauryl ether (Brij35) as the mixed surfactant systems, at a different molar ratio of the two surfactants, the solubility of curcumin in SDS/Brij35 mixed micellar systems was studied by ^1^H NMR [39], 2D DOSY [40] and nuclear Overhauser effect spectroscopy (NOESY) [41]. Due to its specific sensitivity to variations in the molecular chemical environment induced by molecular interactions, NMR has become a powerful technique to study the process and mechanism of micellar solubilization [42,43]. Based on the structural and dynamic information at the atomic and molecular scale provided by NMR spectroscopy, the solubility of curcumin, the formation process and structure of SDS/Brij35 mixed micelles, the solubilization sites of curcumin, as well as the relationship between the solubility and the properties of mixed micelles have been systematically investigated, which are expected to provide new insights into the understanding and optimization of the mixed micellar solubilization systems.

## 2. Results and Discussion

### 2.1. Apparent Solubility and Molar Solubilization Ratio (MSR)

**Figure 1:**
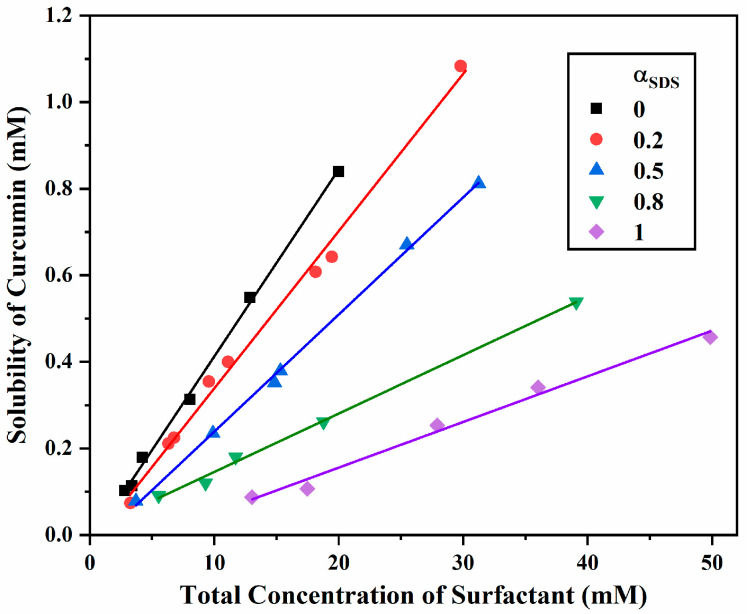
The apparent solubility of curcumin in SDS/Brij35 mixed surfactant systems with different α_SDS_ as a function of C_T_.

The apparent solubility of curcumin in different SDS/Brij35 mixed systems with different α_SDS_ (α_SDS_ = 1.0, 0.8, 0.5, 0.2 and 0.0) as a function of total surfactant concentration (C_T_) is shown in Figure 1. It can be found that the apparent solubility of curcumin increases with the increase in C_T_ in all systems and can be well fitted linearly. 

**Figure 2:**
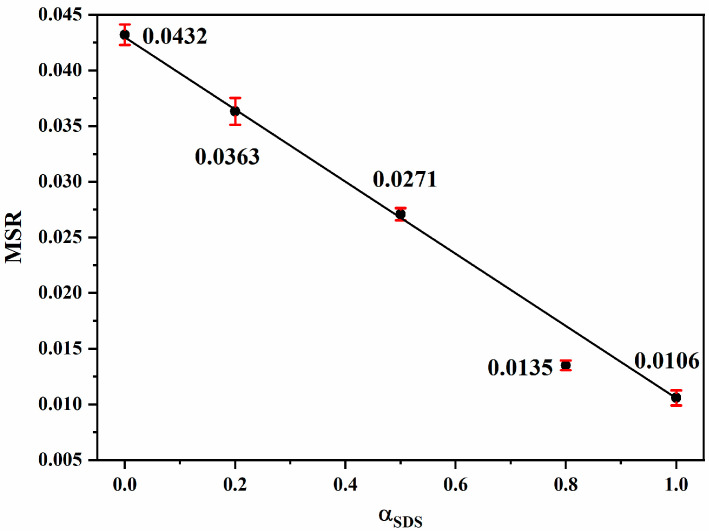
MSRs of curcumin in SDS/Brij35 mixed systems at different molar ratio of SDS (α_SDS_).

By linearly fitting the apparent solubility of curcumin as a function of the surfactant concentration, the slope is the MSR [44], which can be used to assess the solubility of the solubilized substance. The obtained MSRs at different α_SDS_ are shown in Figure 2. In mixed surfactant systems, it has been proposed that the MSR of the solubilized substance can be estimated using the MSR in a single surfactant solution based on the ideal mixing rule [45,46].

(1) $${\mathrm{MSR}}_{\mathrm{ideal}}={\mathrm{MSR}}_{1}X_{1}+{\mathrm{MSR}}_{2}X_{2}+{\mathrm{MSR}}_{\mathrm{water}}$$

where MSR_ideal_ is the calculated MSR by mixed surfactants in the ideal mixed state. MSR_1_ and MSR_2_ are MSRs by a single surfactant, 1 and 2, respectively. X_1_ and X_2_ are molar ratios of surfactants 1 and 2 in mixed surfactant solutions, respectively. MSR_water_ is the MSR in pure water. For curcumin, it is 5.37 × 10^−1^^0^, which can be negligible. Furthermore, the deviation ratio (R) between MSR_exp_ and MSR_ideal_ can be evaluated according to the following equation:

(2) $$R={\mathrm{MSR}}_{\exp}/{\mathrm{MSR}}_{\mathrm{ideal}}$$

when R is greater than 1, this implies that there is a positive mixing effect of mixed surfactants on the solubilization and vice versa.

The MSRs of curcumin in pure Brij35 and SDS are 0.0432 and 0.0106, respectively. The solubility of curcumin in pure Brij35 is significantly greater than that in pure SDS. However, for three SDS/Brij35 mixed systems with α_SDS_ = 0.8, 0.5 and 0.2, the MSR is greater than that of pure SDS but lower than that of pure Brij35. Additionally, we can draw a line by connecting the MSR values in pure SDS and pure Brij35 to obtain a model for MSR_ideal_. For α_SDS_ = 0.5 and 0.2, the obtained MSRs just fall right on the model for MSR_ideal_, indicating that R is equal to 1, there is no positive or negative mixing effect on the solubilization and the MSR depends on the surfactant composition in the system, i.e., the higher the molar ratio of Brij35, the higher the solubility of curcumin, vice versa. However, for α_SDS_ = 0.8, the MSR is significantly less than MSR_ideal_, i.e., R is less than 1. This demonstrates that there is a negative mixing effect on the solubilization. Why is the MSR proportional to the surfactant molar ratio at α_SDS_ = 0.5 and 0.2 and why is there a negative mixing effect at α_SDS_ = 0.8? 

### 2.2. The Formation Process of Mixed Micelles

**Scheme 1:**
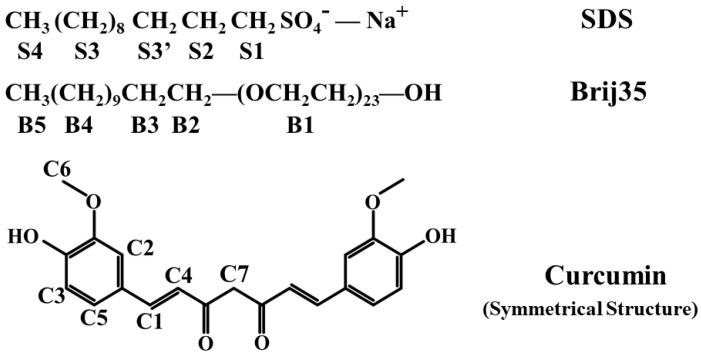
Chemical structures and proton numbering of SDS, Brij35 and curcumin.

**Figure 3:**
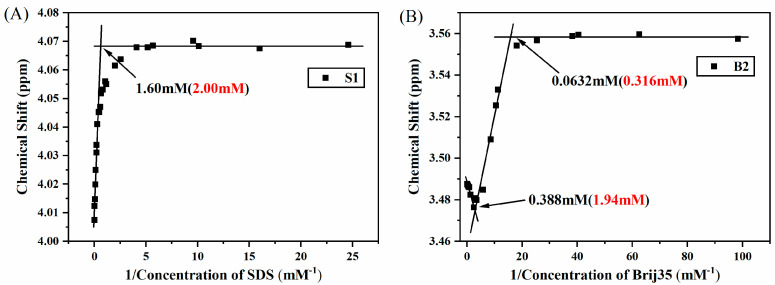
For α_SDS_ = 0.8, variations of chemical shift of the resonance S1 from SDS (**A**) and B2 from Brij35 (**B**) as a function of reciprocals of their concentrations. The intersection of the fitted lines indicates the CMC, the corresponding C_T_ is shown in red font.

To answer the above questions, it may be necessary to understand the formation process of micelles. NMR chemical shift is sensitive to the chemical environment of molecules, allowing us to understand the process of surfactant aggregation and micelle formation. The chemical structures and proton numbering of SDS, Brij35 and curcumin are shown in Scheme 1. Representative ^1^H NMR spectra of SDS/Brij35 in pure and mixed systems are shown in Figure S1. According to the pseudo-phase transition model [47,48], the CMC of SDS and Brij35 in the curcumin-solubilized system at α_SDS_ = 0.8 can be determined by chemical shift changes of resonances S1 and B2 as a function of the reciprocal of their respective concentrations (Figure 3). The obtained CMC of SDS is 1.60 mM, corresponding to a C_T_ of 2.00 mM. Quite different from the chemical shift change of S1, B2 undergoes a process with two distinct phases, first moving to the high field and then to the low field. Likewise, CMCs of Brij35 were obtained. They are 0.0632 mM (designated as CMC_1_) and 0.388 mM (designated as CMC_2_), corresponding to C_T_ of 0.316 and 1.94 mM, respectively. In a study of the mixed system composed of Brij35 and a fluorinated surfactant, a second CMC of Brij35 was reported and attributed to the change in the structure of mixed micelles. With this in mind, the composition or structure of Brij35 aggregates here may also change at CMC_2_. Overall, Brij35 aggregates first and then SDS also starts to aggregate at the C_T_ of 2.00 mM. Interestingly, the CMC_2_ of Brij35 is very close to the CMC of SDS, which makes one wonder if SDS and Brij35 form single mixed micelles at this concentration. 

Similarly, CMC values for SDS and Brij35 were also obtained for α_SDS_ = 0.5 and 0.2 and are shown in Figures S2 and S3 in the Supporting Information. For α_SDS_ = 0.5, the chemical shift change of B2 follows a similar rule to α_SDS_ = 0.8. For α_SDS_ = 0.2, although the chemical shift of B2 always shifts upfield within the studied concentration range, we can still find two phases with different rates of change. CMC values of all mixed systems with different α_SDS_ are shown in Table 1.

For comparison, CMCs in the pure SDS and pure Brij35 systems in the presence of curcumin were also obtained, respectively. As can be seen in Table 1, the addition of Brij35 decreases the CMC of SDS. The greater the amount of Brij35, the greater the decrease in the CMC of SDS. For Brij35, the same rules apply. Furthermore, in our earlier study, it has been found that one component with the smaller CMC value aggregates first [49]. In the present study, for three mixed systems, Brij35 always aggregates first at CMC_1_ and then SDS at higher concentrations because of the inherently larger CMC of SDS than Brij35. This is also consistent with literature reports [49,50]. 

We also performed another surface tension (ST) measurement to characterize the CMC value. Variations in ST in SDS/Brij35 mixed systems solubilized by curcumin as a function of C_T_ are shown in Figure S4. The intersection of two lines indicates the CMC value. From Figure S4, we can find that in all three systems, ST decreases as C_T_ increases. However, the variation trends are different to some extent. For α_SDS_ = 0.5 and 0.8, ST experiences three different stages. In the beginning, ST decreases significantly as C_T_ increases. After that, from a certain concentration, it decreases slowly. In the end, ST arrives on a plateau. By fitting the ST values segmentally, two CMC values were obtained for each system. For α_SDS_ = 0.2, ST also decreases significantly as C_T_ increases at the beginning. Then, from a certain concentration, ST almost keeps constant. Although ST increases slightly as C_T_ approaches 0.922 mM, it can be considered unchanged due to the small range of change. Accordingly, a CMC value of 0.167 mM was obtained. 

To compare with NMR results, CMC values obtained by ST are also shown in Table 1 and marked with the superscript ST. For α_SDS_ = 0.5 and 0.8, we can find that compared to three CMC values obtained by NMR, there are only two CMC values characterized by ST, which are almost consistent with the CMC_1_ and CMC_2_ of Brij35 within the error range, respectively. However, the CMC of SDS was missed by ST. We speculate that due to the higher surface activity of Brij35 than SDS, the decrease in ST is mainly caused by Brij35, hence only manifesting CMC values of Brij35. Whereas, for α_SDS_ = 0.2, different from α_SDS_ = 0.5 and 0.8, there is only one CMC value, which is close to the CMC_1_ of Brij35. Similarly, since the proportion of Brij35 is dominant, the minimum of ST is reached at CMC_1_. It can also be proved by the fact that the variation in the chemical shift of B2 at CMC_2_ is not as significant as those at α_SDS_ = 0.5 and 0.8. In all, CMC values obtained by ST are generally consistent with those by NMR spectroscopy. 

### 2.3. The Composition and Morphology of Micelles

**Figure 4:**
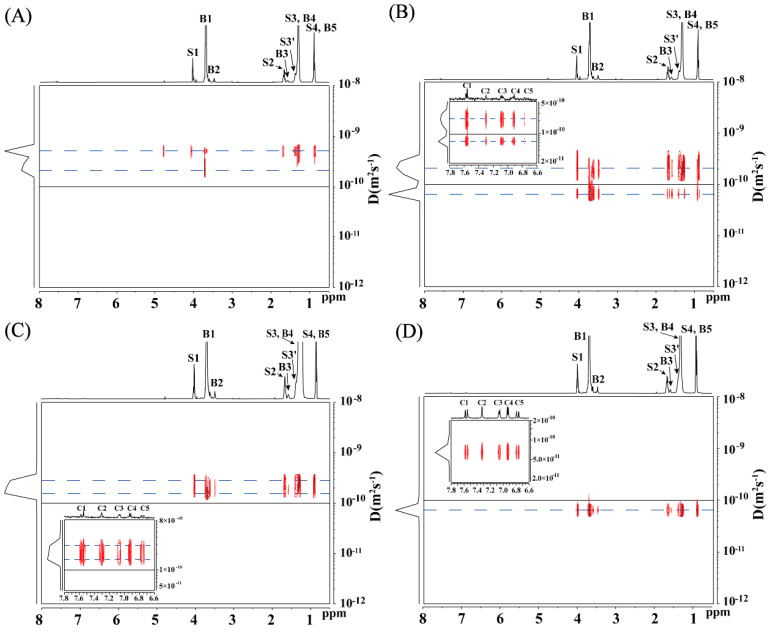
The 2D DOSY plots for the system with α_SDS_ = 0.8 at C_T_ of 0.25 (**A**), 5.0 (**B**), 10 (**C**) and 80 mM (**D**). The black horizontal line at the D value of 10^−10^ m^2^ s^−1^ is a reference to mark the variation of the diffusion coefficients.

To explore the changes in micelle composition and morphology during the solubilization, 2D DOSY experiments were further performed. The 2D DOSY spectra of the α_SDS_ = 0.8 system with a C_T_ range from 0.25 to 80 mM are shown in Figure 4 and Figure S5. The ^1^H NMR spectrum and the diffusion dimension projection are shown at the top and left of the 2D DOSY spectrum, respectively. The D value of 10^−10^ m^2^ s^−1^ along the diffusion dimension is marked by a black horizontal line to indicate the change in the self-diffusion coefficients.

At C_T_ of 0.25 mM, there are two different diffusion coefficients in the diffusion dimension (Figure 4A). Based on their chemical shifts, it can be inferred that they belong to SDS and Brij35, with values of 5.13×10^−10^ and 2.12×10^−10^ m^2^ s^−1^, respectively. Furthermore, since the current concentration is lower than the CMCs of Brij35 and SDS, we speculate that these two diffusion coefficients originate from the SDS and Brij35 molecules in the monomeric state. Figure S5 A shows the 2D DOSY spectrum at C_T_ of 0.50 mM (corresponding to the Brij35 concentration of 0.10 mM, slightly above its CMC_1_). The diffusion coefficient of SDS remains at 5.13×10^−10^ m^2^ s^−1^, indicating that SDS molecules are still in the monomeric state. Although it can be inferred from the change in the chemical shift that Brij35 starts to aggregate, its diffusion coefficient remains unchanged. This may be due to the lower surfactant concentration, the movement of Brij35 in aggregates is relatively free. 

When C_T_ is 2.0 mM, close to the CMC_2_ of Brij35 and the CMC of SDS, the diffusion coefficients of SDS and Brij35 decrease to 3.81 ×10^−10^ m^2^ s^−1^ and 1.17×10^−10^ m^2^ s^−1^, respectively (Figure S5B). According to the Stokes–Einstein diffusion law, the decrease in diffusion coefficient indicates the size of Brij35 aggregates is larger. Combined with the CMC obtained from chemical shift changes, it can be concluded that SDS molecules also begin to aggregate. Although the CMC (2.00 mM) of SDS is almost equal to the CMC_2_ (1.94 mM) of Brij35, SDS and Brij35 do not form single mixed micelles. They diffuse at different diffusion coefficients. We previously noticed the unusual change in the chemical shift of B2 at CMC_2_. Combined with the change in diffusion coefficient here, we speculate that SDS molecules are involved in the formation of Brij35 aggregates at CMC_2_ and the electron-withdrawing effect of the polar head of SDS probably results in the downfield shifting in the chemical shift of B2. Likewise, the significantly lower CMC of SDS in the mixed system is probably due to the intermolecular interaction between SDS and Brij35. Therefore, the formed SDS micelles are also doped with a small amount of Brij35 molecules. Vinarov et al. reported that there were separate two kinds of micelles formed in bile salt and surfactant mixed systems and named them “bile salt-rich micelles” and “surfactant-rich micelles” [31]. The micellar types and structures in their study are very similar to those in our study. Therefore, we also name our two mixed micelles “SDS-rich micelles” and “Brij35-rich micelles”, respectively. It was reported that only a small fraction of foreign molecules coexisted in the mixed system of NaTDC and some nonionic surfactants in individual micelles, i.e., <10% [31]. In our study, we speculate that the proportion of foreign molecules in SDS/Brij35 mixed micelles is also low. On the one hand, similar to the NaTDC mixed system, ^1^H signals of foreign molecules are also not significantly resolved in DOSY spectra. On the other hand, since the molar ratio of Brij35 is 0.2, if the proportion of Brij35 in the SDS-rich micelles reaches 20%, SDS and Brij35 will form a single mixed micelle. In addition, SDS-rich micelles with the larger diffusion coefficient are smaller in size, thus diffusing faster than Brij35-rich micelles. Additionally, in the diffusion dimension, the signal corresponding to SDS-rich micelles is stronger than that corresponding to Brij35-rich micelles due to the higher molar proportion in the mixed system. 

When C_T_ increases to 5 mM (Figure 4B), both D values continue to decrease, with diffusion coefficients of 2.12 and 0.64 × 10^−10^ m^2^ s^−1^, respectively, indicating further growth in their size. Furthermore, at this concentration, clear curcumin peaks can be observed from the ^1^H NMR spectrum. In 2D DOSY, most curcumin molecules co-diffuse with SDS-rich micelles. Additionally, a small amount of curcumin is also co-diffused with Brij35-rich micelles. These indicate that curcumin is not only solubilized in SDS-rich micelles but also in Brij35-rich micelles. It is worth noting that in pure SDS solution at a concentration of 5 mM, ^1^H signals of curcumin are barely visible, not to mention co-diffusion with SDS molecules. This is another evidence demonstrating the mixed micellar structure of SDS-rich micelles. In addition, At C_T_ = 10 mM (Figure 4C), the diffusion coefficients of SDS and Brij35 unexpectedly increase to 2.42 and 1.59 × 10^−10^ m^2^ s^−1^, respectively, indicating that Brij35-rich and SDS-rich micelles may reconstitute into smaller micelles. Furthermore, in the diffusion dimension, the signal of Brij35 tends to fuse with that of SDS. Therefore, we speculate that the size adjustment of micelles prepares for the fusion of two separate micelles. At C_T_ = 20 and 40 mM (Figure S5C,D), the two diffusion coefficients decrease again and the difference between them also becomes smaller, indicating that SDS and Brij35 micelles are fusing. At C_T_ = 80 mM (Figure 4D), there is only one distinct signal in the diffusion dimension with a D value of 0.64 × 10^−10^ m^2^ s^−1^, indicating that Brij35-rich and SDS-rich micelles are completely fused to form a single SDS/Brij35 mixed micelle. The decrease in the D value indicates the increase in the micellar size. Besides, curcumin is solubilized in single-mixed micelles. 

Representative 2D DOSY spectra for α_SDS_ = 0.5 are shown in Figure S6. When C_T_ is 0.20 mM (corresponding to the concentration of 0.10 mM for both SDS and Brij35, between the CMC_1_ of Brij35 and the CMC of SDS), only one D value of 2.83 × 10^−10^ m^2^ s^−1^ is observed in the diffusion dimension (Figure S6A). Although some resonances of SDS are too weak or overlapped with those of Brij35, it can still be determined that this diffusivity comes from both Brij35 and SDS. This suggests that SDS molecules also join them when Brij35 aggregates are formed at CMC_1_. In addition, the diffusion coefficient of 2.83 × 10^−10^ m^2^ s^−1^ is between 5.13 × 10^−10^ m^2^ s^−1^ and 2.12 × 10^−10^ m^2^ s^−1^, corresponding to the individual diffusion coefficients of SDS and Brij35 in the monomeric state in the system with α_SDS_ = 0.8. Therefore, we speculate that Brij35 and SDS aggregate into small aggregates (oligomers). When C_T_ equals 2.00 mM (Figure S6B), which is higher than the CMC (0.384 mM, corresponding to C_T_ of 0.768 mM) of SDS, it can be found that SDS still co-diffuses with Brij35. However, the diffusion coefficient remains unchanged, indicating that the aggregates do not become larger. This may also be caused by the free movement of surfactant molecules due to the loose structure of oligomers. As the concentration continues to increase (Figure S6C,D), SDS and Brij35 always diffuse together, but the diffusion coefficient decreases, suggesting the size of the aggregates become larger. Moreover, resonances from curcumin can also be observed in 2D DOSY spectra in Figure S6C,D, which proves that curcumin is solubilized in the mixed micelles.

**Figure 5:**
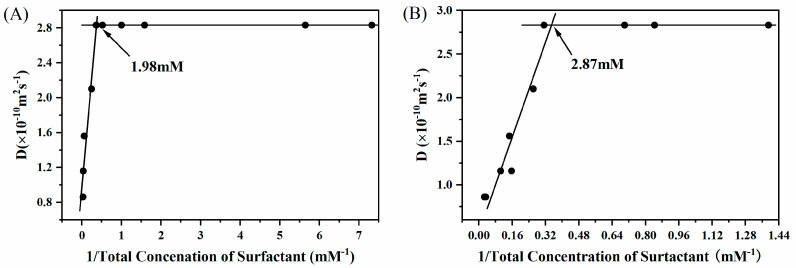
For α_SDS_= 0.5 (**A**) and 0.2 (**B**), variations of diffusion coefficients of the mixed micelles as a function of the reciprocal of C_T_.

By fitting the variations in diffusion coefficient with the reciprocal of C_T_, we also obtained the CMC value, which is consistent with the CMC_2_ obtained from the variations in chemical shift of B2 (Figure 5A). In our previous discussion, we speculated that the downfield shift of B2 above CMC_2_ of Brij35 was related to changes in the composition of aggregates. Combined with the change in the diffusion coefficient, we infer that the mixed oligomers start to aggregate into mixed micelles at this concentration. During this process, the proportion of SDS in the mixed aggregates increases, resulting in the downfield shift of B2. After reconstitution, the mixed micelles continue to grow larger with the increase in C_T_, resulting in slower diffusion. 

For α_SDS_ = 0.2, similar to the system with α_SDS_ = 0.5, there is also only one sharp resonance along the diffusion dimension over the entire concentration range from 0.045 to 40 mM. Figure S7A shows the 2D DOSY spectrum at C_T_ = 0.20 mM, between the CMC_1_ and CMC_2_ of Brij35 and higher the CMC of SDS. The diffusion coefficient is 2.83 × 10^−10^ m^2^ s^−1^, which is the same as that of the system with α_SDS_ = 0.5 at low concentrations. At a high concentration of 20 mM (Figure S7B), higher than the CMCs of SDS and Brij35, the diffusion coefficient decreases to 1.16 × 10^−10^ m^2^ s^−1^. According to the resonance assignment, SDS and Brij35 always diffuse together, suggesting single mixed micelles are formed. In addition, curcumin is also solubilized in the mixed micelles. By fitting the diffusion coefficient as the reciprocal of C_T__,_ the obtained CMC value (2.30 mM, corresponding to C_T_ of 2.87 mM) is also consistent with the CMC_2_ obtained from the chemical shift change (Figure 5B). Therefore, the same conclusion obtained from the α_SDS_ = 0.5 system can be deduced in this system, that is, above the CMC_1_ and CMC_2_ of Brij35, Brij35 and SDS aggregate into mixed oligomers and micelles, respectively. Furthermore, at low surfactant concentrations, the diffusion coefficients of the oligomers for α_SDS_ = 0.5 and 0.2 are the same. We deduce that they are almost the same oligomers dominated by Brij35. This is mainly because of the following: firstly, Brij35 has a lower CMC and a stronger aggregation capability than SDS; secondly, in both systems, Brij35 has an equipotent or dominant molar ratio. 

### 2.4. The Micellar Structure and Solubilization Sites of Curcumin

The above results indicate that there are different processes of micelle formation and curcumin solubilization. To obtain the structure of the micelles and solubilization sites of curcumin, we collected their NOESY spectra and the spectra of pure SDS and Brij35 micelles for comparison. In the NOESY spectrum of SDS at a concentration of 100 mM (well above its CMC), the positive (the same sign as the diagonal peaks) cross peak of S1–S3 demonstrates the formation of SDS micelles (Figure S8). Likewise, for those Brij35 protons that are not spatially adjacent, there are also cross peaks, such as B1–B4 and B1–B5, indicating the formation of micelles (Figure S9). It has been reported that non-ionic TX-100 micelles adopt a spherical, onion-like structure [52,53]. According to cross peaks B1–B4 and B1–B5, Brij35 molecules may also tend to form multilayer micelles in a staggered manner to reduce the electrostatic repulsion between polar heads. 

**Figure 6:**
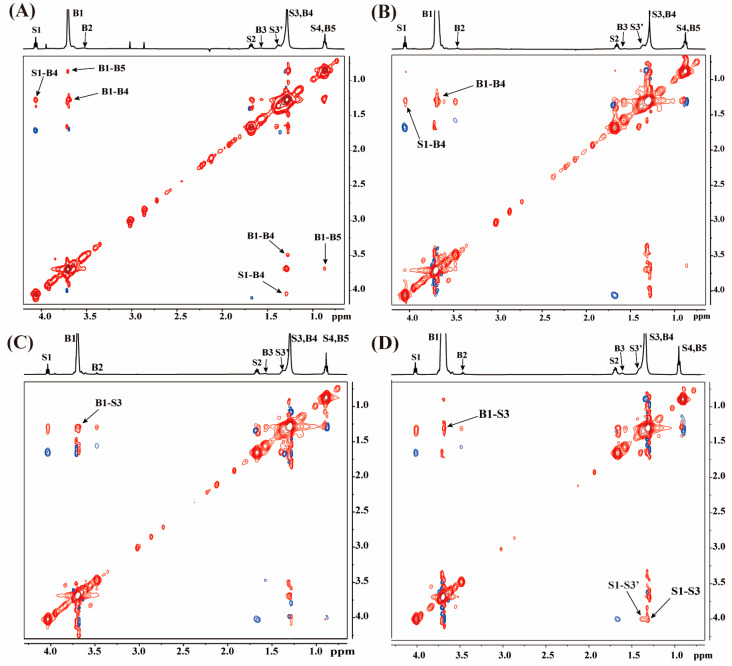
NOESY spectra with a mixing time of 0.5 s for α_SDS_ = 0.8 at C_T_ of 0.50 (**A**), 2.0 (**B**), 5.0 (**C**) and 40 mM (**D**), respectively.

Several representative NOESY spectra of the SDS/Brij35 mixed system with α_SDS_ = 0.8 are shown in Figure 6. At C_T_ = 0.5 mM (Figure 6A), intermolecular cross peaks B1–B4 and B1–B5 can be found. Combined with the obtained CMC_1_, we conclude that small aggregates or oligomers of Brij35 are formed. Besides, the cross peak of S1–S3 or S1–B4 can be observed. Because the resonances of S3 and B4 overlap with each other, we need to determine whether they originate from S1–S3 or S1–B4. Since the concentration of SDS is still lower than its CMC, we deduce that the cross peak is not from S1–S3, but from S1–B4. This suggests that when Brij35 forms oligomers, a few SDS molecules participate in them, forming mixed oligomers of Brij35 and SDS. When C_T_ = 2 mM, close to the CMC_2_ of Brij35 and CMC of SDS, the cross-peak information is almost the same as those at C_T_ = 0.5 mM, but the intensity becomes stronger with the increase in C_T_ (Figure 6B). This suggests that the interaction between SDS and Brij35 becomes stronger, proving the mixed micelle structure of SDS-rich and Brij35-rich micelles. At the same time, the cross peak B1–B5 disappears, indicating that the interaction between Brij35 molecules is weaker. 

**Figure 7:**
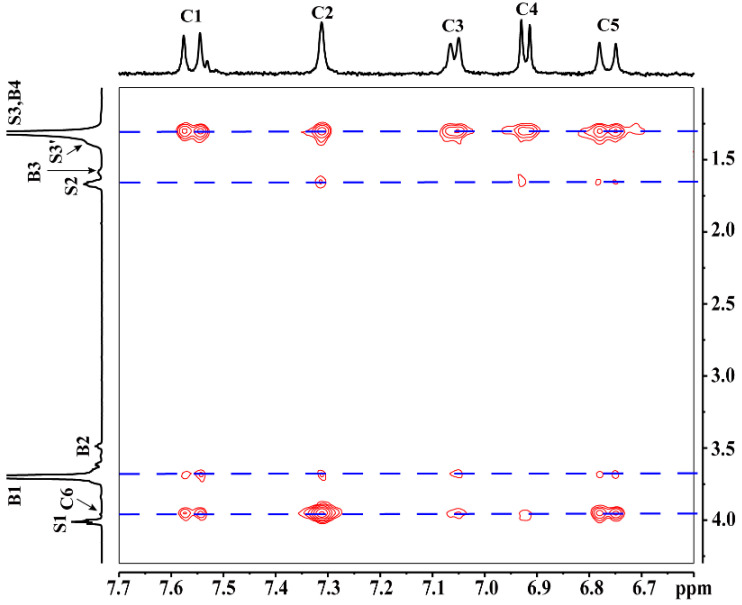
Partially enlarged NOESY spectrum with a mixing time of 0.5 s for α_SDS_ = 0.8 at C_T_ of 40 mM.

At C_T_ of 5 mM, both higher than the CMC_2_ of Brij35 and the CMC of SDS, the cross peak B1–B5 is still not shown up, further indicating a weak interaction between Brij35 molecules (Figure 6C). When C_T_ = 40 mM, the 2D DOSY spectrum shows that Brij35-rich micelles almost fuse with SDS-rich micelles to form single mixed micelles. In the NOESY spectrum (Figure 6D), there are clear cross peaks S1–S3 and S1–S3’. In addition, the line shape of cross peak S1-B4/S3 is broadened compared to C_T_ = 5 mM (Figure 6C). These are strong evidences for the formation of SDS micelles. However, the possibility of S1–B4 is not ruled out. In fact, S1 and B4 may be spatially adjacent based on the cross peak B1–S3′. These cross peaks indicate that the hydrophilic group B1 of Brij35 and the hydrophobic chain of SDS, the polar head group S1 of SDS and the hydrophobic chain group B4 of Brij35 are close to each other. Brij35 molecules possibly dope into SDS micelles to reduce electrostatic repulsion between the polar heads of SDS molecules themselves, and between them and the polyoxyethylene groups of Brij35. In addition, the partially enlarged NOESY spectrum containing the cross-peak information of curcumin at C_T_ = 40 mM is shown in Figure 7. Cross peaks between curcumin proton groups and S3/B4 and B1 indicate that curcumin is solubilized at the interaction sites of two surfactants. 

For α_SDS_ = 0.5, NOESY spectra at C_T_ of 1 and 16 mM are shown in Figure S10. When C_T_ is 1 mM (Figure S10A), between CMC_1_ and CMC_2_ of Brij35 and higher than the CMC of SDS. Similar to the system with α_SDS_ = 0.8, there are also indistinguishable cross peaks S1–S3/B4 and B1–S3/B4. Combined with the conclusion deduced before that SDS/Brij35 mixed oligomers are formed at this concentration, it can be determined that these cross peaks come from S1–B4 and B1–S3. In addition, the presence of the cross peak B1–B5 indicates that Brij35 molecules in mixed micelles are close to each other due to the higher molar ratio of Brij35, suggesting a multilayered structure similar to that of pure Brij35 micelles. At C_T_ of 16 mM (Figure S10B), it is significantly higher than CMC_2_ of Brij35. Although there are also indistinguishable S1–S3/B4 and B1–S3/B4, the cross peak between B1 and S2 demonstrates the formation of mixed micelles. Compared to the absence of B1–B5 at higher concentrations for α_SDS_ = 0.8, the presence of B1–B5 indicates an increased chance of intermolecular interactions between Brij35 molecules as α_Brij35_ increases. 

NOESY spectra for α_SDS_ = 0.2 at C_T_ of 2 and 20 mM are shown in Figure S11. Compared to α_SDS_ = 0.5, the cross-peak information is not much different. Due to the increased molar ratio of Brij35, even if the C_T_ is merely 2 mM (Figure S11 A), which is between the CMC_1_ and CMC_2_ of Brij35 and higher than the CMC of SDS, the cross peak B1–B5 shows up, indicating a stronger interaction between Brij35 molecules. At higher concentrations (Figure S11B), there is also the cross peak B1–S2, but with a weaker intensity, demonstrating a weaker interaction between SDS and Brij35 because of the decreased molar ratio of SDS. Similar cross peaks between curcumin and surfactants in the system with α_SDS_ = 0.8 can also be found in systems with α_SDS_ = 0.5 and 0.2 in Figure S12 and Figure S13, indicating that solubilization sites of curcumin are the same in three systems. 

### 2.5. The Surfactant Aggregation Process (CMC) and the Solubilization of Curcumin 

It should be noted that for these three mixed systems, the aggregation of SDS and Brij35 occurs at low surfactant concentrations, typically below 2 mM. From the solubilization results, the solubility of curcumin at this time is too low to be detected in ^1^H NMR spectra. Therefore, on one hand, the effect of the solubilization of curcumin on the aggregation (CMCs) of SDS and Brij35 can be ignored. On the other hand, the aggregation process of surfactants also has no effect on the solubilization of curcumin. 

### 2.6. The Composition of Mixed Surfactants and the Micellar Structure

However, at higher surfactant concentrations (C_T_), typically above 2 mM, resonances of curcumin are observable in NMR spectra. Based on the co-diffusion of curcumin and surfactants in 2D DOSY spectra, we conclude that curcumin is solubilized in the formed surfactant micelles. This means that the solubility of curcumin depends on the type and structure of surfactant micelles. In this study, SDS and Brij35 in mixed systems with α_SDS_ = 0.5 and 0.2 aggregate into single mixed oligomers at low concentrations and single mixed micelles at high concentrations. For α_SDS_ = 0.8, mixed oligomers of Brij35 and SDS are also formed above the CMC_1_ of Brij35. However, separate mixed micelles form then and are present over a wide range of concentrations. Single mixed micelles finally form at a C_T_ of 80 mM. 

Different micelle formation processes and micelle structures are closely related to the different molecular sizes and properties of surfactants. It is known that the molecular size of Brij35 is larger than that of SDS. According to the Stokes-Einstein equation, the hydrodynamic radii of kinetic units of SDS and Brij35 in aqueous solutions at varied concentrations for α_SDS_ = 0.8 were calculated according to the diffusion coefficients and are shown in Table S1. At low concentrations of 0.25 and 0.50 mM, the hydrodynamic radii of kinetic units of SDS are 0.430 nm, corresponding to the effective hydrodynamic radii of SDS monomers, which is very close to the literature data of 0.35 nm [54]. From the size of two kinds of aggregates (0.573 and 1.87 nm at C_T_ = 2 mM, 1.00 and 3.31 nm at C_T_ = 5 mM), the size of the Brij35-rich micelles is about three times that of the SDS-rich micelles. When C_T_ is 10 mM, the size of two micelles becomes smaller and the difference between them also becomes smaller. Additionally, as C_T_ increases, the size difference between the two micelles gets smaller and smaller until they are identical (the micelles fuse together). From this point of view, they tend to be separate micelles when the micelle size difference is large and single mixed micelles when the micelle size difference is small. In other words, the mutual hindrance of small and larger micelles prevents the fusion of micelles. Specifically, SDS-rich micelles dominate due to the higher SDS concentration. Additionally, the small size of SDS-rich micelles hinders the fusion of larger Brij35 micelles to form single mixed micelles. However, for the system with α_SDS_ ≤ 0.5, Brij35 is dominant and its larger micelles can encapsulate smaller SDS molecules to form stable mixed micelles. Furthermore, the repulsive force between the polar heads of SDS and the hydrophilic polyoxyethylene groups may be another obstacle to the formation of single mixed micelles. 

### 2.7. The Micellar Morphology and the Solubilization Sites 

Besides the composition of surfactants in the mixed system, another factor that may affect the solubility of curcumin is the micellar morphology and solubilization sites of curcumin in micelles. From the above discussion, in these three mixed systems, curcumin is always solubilized in mixed micelles. Although cross peaks between SDS and Brij35 or between themselves are strong or weak based on their molar ratios in mixed systems, cross-peak types are nearly identical, suggesting that SDS and Brij35 may have similar interaction modes and sites despite different molar ratios. Furthermore, the solubilization sites of curcumin in these mixed micelles are also the same. 

### 2.8. The Molar Ratio of Mixed Surfactants and Solubility

We know that for α_SDS_ = 0.5 and 0.2, curcumin is always solubilized in the single mixed micelles. From the above discussion, due to their similar mixed micellar structures and solubilization sites, the solubility of curcumin may only depend on the molar ratio of SDS or Brij35 in mixed surfactants. As for what proportion of Brij35 or SDS would satisfy this rule, it cannot be determined in this study. However, when the proportion of Brij35 is equal to or greater than 50%, it can be said that this rule is true. 

For α_SDS_ = 0.8, MSR is evidently lower than the MSR_ideal_ model. Although the positive mixing effect of anionic-nonionic mixed surfactant systems has been reported in some studies [20,44], it has also been reported that the solubilization capability of mixed surfactant systems is between those of two pure surfactant solutions [55,56], and even the solubility drops down to a negligible value due to the precipitation when anionic and cationic surfactants are mixed in equimolar doses [57]. Therefore, we deduce that unlike the other two systems, because the α_SDS_ = 0.8 system cannot form single mixed micelles, the MSR at α_SDS_ = 0.8 is significantly lower than the MSR_ideal_ model. We speculate that the main reasons are the following: firstly, SDS-rich micelles are smaller in size compared to Brij35-rich micelles; secondly, the concentration of SDS-rich micelles is much higher than that of Brij35-rich micelles; finally, the repulsive force between the polar head of SDS and the hydrophilic group of Brij35 may also prevent Brij35 from joining SDS micelles to form single mixed micelles. Furthermore, as we can see, during the fusion process, two separate mixed micelles recombine into single mixed micelles. This suggests that the structure of these two separate micelles is unstable, which may be the reason for the low solubility of this system.

## 3. Materials and Methods

### 3.1. Materials

SDS (MW = 288.38, 98%) and curcumin (MW = 368.37, 96%) were purchased from Sigma-Aldrich (Shanghai, China) Trading Co., Ltd. Brij35 (MW = 1198, AR) and D_2_O (99.9% deuterium) were purchased from Qingdao Tenglong Weibo Technology. All reagents mentioned above were employed directly as received, without any purification. Instead of water, D_2_O was used as solvent to weaken the water signal.

### 3.2. Sample Preparation

For NMR measurement, first, a series of D_2_O solutions of mixed surfactants of SDS and Brij35 were prepared with the molar ratio of SDS (α_SDS_) equaling 0.8, 0.5 and 0.2. The total concentrations (C_T_) of mixed solutions vary according to the requirements at each molar ratio. Then, multiple portions of excessive curcumin (10 mg of each) were weighted and added into each surfactant solutions with a volume of 700 μL. Next, the mixed solutions with curcumin added were oscillated at 200 rpm in a reciprocating water bath oscillator at 25 °C for 48 h. After oscillation, to separate undissolved curcumin, samples were centrifuged at 10,612 g for 10 min. Finally, each of 600 μL supernatant was transferred into the 5 mm NMR tube for further experiments. For comparison, the samples with the single surfactant SDS or Brij35 were also prepared according to the same procedure.

For surface tension and pH measurements, sample preparation protocols are the same as for NMR experiments. The only difference is that the volume of each sample is 8 mL and 114 mg of curcumin is added accordingly to ensure the same ratio of solute to solvent. At the same time, the surfactant concentration remains consistent. 

### 3.3. NMR Experiments

All NMR experiments were performed at 25 °C using a 500 MHz Agilent ProPulse NMR spectrometer (Agilent Technologies, Santa Clara, CA, USA) equipped with a 5 mm XYZ indirect detection probe and Z-direction gradient coils with a maximum nominal gradient strength of 60 G cm^−1^. For assuring the complete recovery of magnetization vector, a small pulse flip-angle 30° was used rather than 90° in ^1^H NMR spectroscopy.The solution of TSP (Me_3_Si-CD_2_CD_2_-CO_2_Na) of known concentration in a capillary was used as the external reference to calibrate the frequency and calculate the actual concentration of the solute and apparent solubility of curcumin. 

The bipolar pulse pair longitudinal eddy-current delay (BPPLED) sequence was used as the 2D DOSY pulse sequence. Typical parameters were as follows: a spectral width of 8 kHz with 16 accumulations, a relaxation delay of 3.2 s with a diffusion delay of 0.2 s, a gradient pulse interval of 0.003 s with the gradient strength linearly ranging from 1 to 36 Gauss cm^−1^ in 32 steps to achieve better signal attenuation. The standard three-pulse sequence was used for NOESY experiments. A mixing time of 0.5 s with a total of 32 accumulations, 4 k and 256 sampling data for the direct (t_2_) and indirect dimension (t_1_), respectively. The data point array 8 k (F_2_) × 1 k (F_1_) was used after Fourier transformation and the zero filling. All NMR data were processed using MestReNova software (version 14.0.0, Mestrelab Research, Santiago de Compostella, Spain). 

### 3.4. Surface Tension and pH Measurements

The CMC value was also determined by surface tension measurement using an automatic tensiometer (QBZY-1, Fangrui, Shanghai, China) at 25 ± 0.1°C. Surface tension values for each sample are given as the mean ± SD (standard derivation) of multiple measurements. The measured pH values for all samples are approximately 7.2 ± 0.1.

## 4. Conclusions

In summary, the solubilization of curcumin by mixed micellar systems of SDS and Brij35 at different surfactant molar ratios was investigated by a series of NMR methods. First, the MSRs of each mixed surfactant system (α_SDS_ = 0.8, 0.5 and 0.2) are between those of pure Brij35 (the maximum MSR) and pure SDS (the minimum MSR) systems. For α_SDS_ = 0.5 and 0.2, MSR is equal to MSR_ideal_ and depends on the molar ratio of surfactant. However, for α_SDS_ = 0.8, the solubility is slightly weakened compared to MSR_ideal_. 

For all three systems, the formation of mixed micelles is a multi-step process. Brij35 with the smaller CMC always aggregates first, followed by SDS at higher concentrations. At even higher concentrations, the mixed oligomers continue to aggregate into mixed micelles. During this process, no solubilization of curcumin is detected due to the low solubility. Thus, the process of surfactant aggregation is not affected by the solubilization of curcumin and vice versa. 

2D DOSY shows that curcumin is solubilized in mixed micelles. This suggests that the solubility depends on the structure of the mixed micelles. For α_SDS_ = 0.5 and 0.2, curcumin is solubilized in single mixed micelles. NOESY spectra show that the mixed micellar structure is similar and the solubilization sites of curcumin are the same. Therefore, the solubility of curcumin may only depend on the molar ratio of surfactant in the mixed system. For α_SDS_ = 0.8, although similar cross-peak information was obtained, however, there are two separate mixed micelles. The low solubility of the system indicates that the solubilization capacity of these two separate mixed micelles is lower than that of single mixed micelles. These results demonstrate that different types of mixed micelles may form based on the different molar ratios of surfactant, which is associated with the properties of surfactants, further resulting in different solubilization capacities.

In conclusion, the intrinsic factors influencing the solubilization capacity of curcumin in mixed micelles of SDS and Brij35 were revealed from the investigation of the micelle formation process, mixed micelle structure and solubilization sites. This study not only provides a systematic research scheme for the mechanism study of the solubilization of poorly soluble solutes in mixed micelles but also provides new insights into the relationship between the solubility and micelle formation process, type and structure of mixed micelles and solubilization sites. 

## Figures and Tables

**Table 1:** CMC values of SDS and Brij35 in mixed systems at different molar ratios of SDS (α_SDS_).

	SDS	Brij35	
α_SDS_	CMC (C_T_) /mM	CMC_1_ (C_T_) /mM	CMC_2_ (C_T_) /mM
1	6.91 (8.1 mM ^a^)		
0.8	1.60 (2.00)	0.0632(0.316, 0.254 ^ST^)	0.388 (1.94, 1.92 ^ST^)
0.5	0.384(0.768)	0.0611(0.122, 0.173 ^ST^)	0.916(1.83, 1.82 ^ST^)
0.2	0.0532(0.266)	0.131(0.164, 0.167 ^ST^)	2.15(2.69)
0		0.240 (0.066 mM ^b^)	

^a^ represents the CMC value of pure SDS [51]. ^b^ represents the CMC value of pure Brij35 [45]. ^ST^ represents the CMC value determined by surface tension.

## Data Availability

Data is contained within the article or supplementary material.

## Supplementary Materials

The following supporting information can be downloaded at: https://www.mdpi.com/article/10.3390/molecules27155032/s1. Figure S1: Representative ^1^H NMR spectra of SDS/Brij35 in pure Brij35 (A), α_SDS_ = 0.2 (B), α_SDS_ = 0.5 (C), α_SDS_ = 0.8 (D) and pure SDS (E) at the same total concentration of 4 mM, Figure S2: For α_SDS_ = 0.5, variations of chemical shift of the resonance S1 from SDS (A) and B2 from Brij35 (B) as a function of reciprocals of their concentrations. The intersection of the fitted lines indicates the CMC, the corresponding C_T_ is shown in red font, Figure S3: For α_SDS_ = 0.2, variations of chemical shift of the resonance S1 from SDS (A) and B2 from Brij35 (B) as a function of reciprocals of their concentrations. The intersection of the fitted lines indicates the CMC, the corresponding C_T_ is shown in red font, Figure S4: For α_SDS_ = 0.2 (A), 0.5 (B) and 0.8 (C), variations in surface tension in SDS/Brij35 mixed systems solubilized by curcumin as a function of the total concentration of surfactant (C_T_). The intersection of two lines indicates the CMC, Figure S5: 2D DOSY plots for the system with α_SDS_ = 0.8 at C_T_ of 0.50 (A), 2.0 (B), 20 (C) and 40 mM (D). The black horizontal line at the D value of 10^−10^ m^2^ s^−1^ is a reference to mark the variation of the diffusion coefficients, Figure S6: 2D DOSY plots for α_SDS_ = 0.5 at C_T_ of 0.20 (A), 2.0 (B), 8.0 (C) and 64 mM (D). The black horizontal line at the D value of 10^-10^ m^2^ s^-1^ is a reference to mark the variation of the diffusion coefficients, Figure S7: 2D DOSY plots for α_SDS_ = 0.2 at C_T_ of 0.20 (A) and 20 mM (B). The black horizontal line at the D value of 10^−10^ m^2^ s^−1^ is a reference to mark the variation of the diffusion coefficients, Figure S8: NOESY spectrum of the pure SDS solution at a concentration of 100 mM with a mixing time of 1 s, Figure S9: NOESY spectrum of the pure Brij35 solution at a concentration of 2.52 mM with a mixing time of 0.5 s, Figure S10: For α_SDS_ = 0.5, NOESY spectra with a mixing time of 0.5 s at C_T_ of 1.0 (A) and 16 mM (B), Figure S11: For α_SDS_ = 0.2, NOESY spectra with a mixing time of 0.5 s at C_T_ of 2.0 (A) and 20 mM (B), Figure S12: For α_SDS_ = 0.5, at C_T_ of 16 mM, partially enlarged NOESY spectrum with a mixing time of 0.5 s, Figure S13: For α_SDS_ = 0.2, at C_T_ of 20 mM, partially enlarged NOESY spectrum with a mixing time of 0.5 s, Table S1: For α_SDS_ = 0.8, hydrodynamic radii of kinetic units of SDS and Brij35 in aqueous solutions at varied concentrations, the solution viscosity is also shown.
