# Supplementary material for: Mechanism of the Micellar Solubilization of Curcumin by Mixed Surfactants of SDS and Brij35 via NMR Spectroscopy
Source: Molecules. 2022 Aug 8;27(15):5032. doi: 10.3390/molecules27155032 (PMC9370735; doi:10.3390/molecules27155032)
Supplement: Supplementary file 1 [file molecules-27-05032-s001.zip › molecules-1839437-supplementary.pdf]

Supporting Material

Mechanism of the Micellar Solubilization of Curcumin by Mixed Surfactants of SDS and Brij35 via NMR spectroscopy

Xiao Zhan, Zhaoxia Wu, Zhong Chen and Xiaohong Cui *

Fujian Provincial Key Laboratory of Plasma and Magnetic Resonance, State Key Laboratory of Physical Chemistry of Solid Surfaces, Department of Electronic Science, Xiamen University, Xiamen, 361005, China

* Correspondence: cuixh@xmu.edu.cn Tel.: +86-0592-2181681

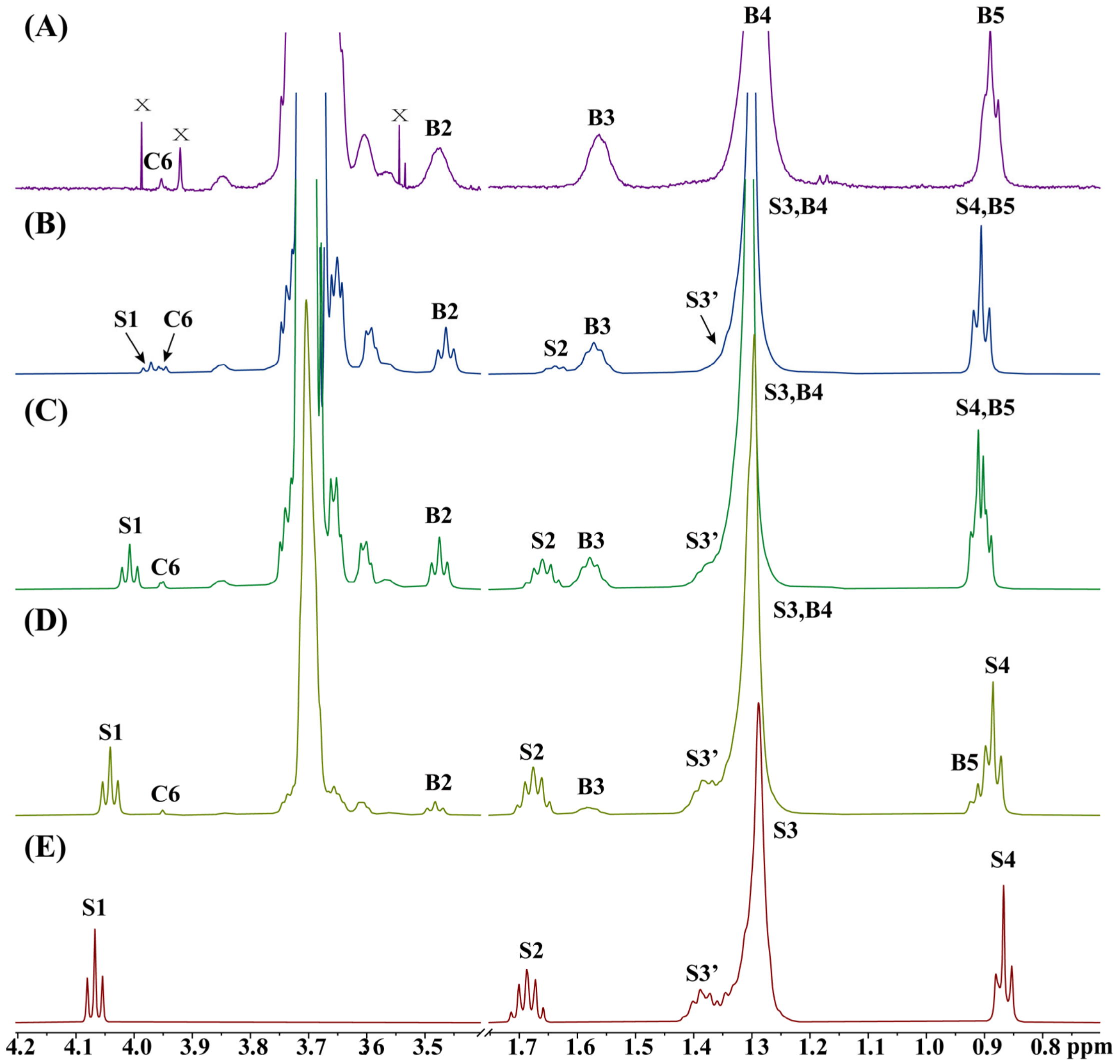

Figure S1 Representative ^{1}H NMR spectra of SDS/Brij35 in pure Brij35 (A), α_{SDS}=0.2 (B), α_{SDS}=0.5 (C), α_{SDS}=0.8 (D) and pure SDS (E) at the same total concentration of 4 mM.

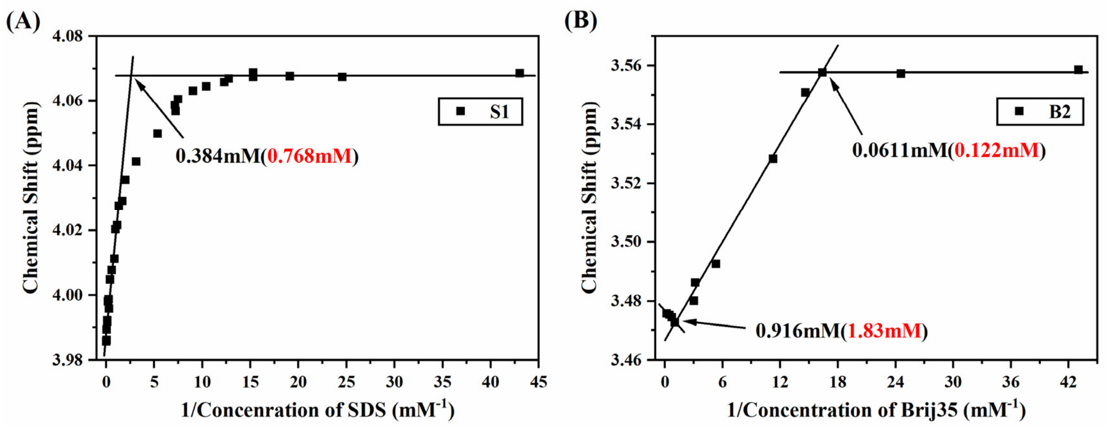

Figure S2 For α_{SDS}=0.5, variations of chemical shift of the resonance S1 from SDS (A) and B2 from Brij35 (B) as a function of reciprocals of their concentrations. The intersection of the fitted lines indicates the CMC, the corresponding C_T is shown in red font.

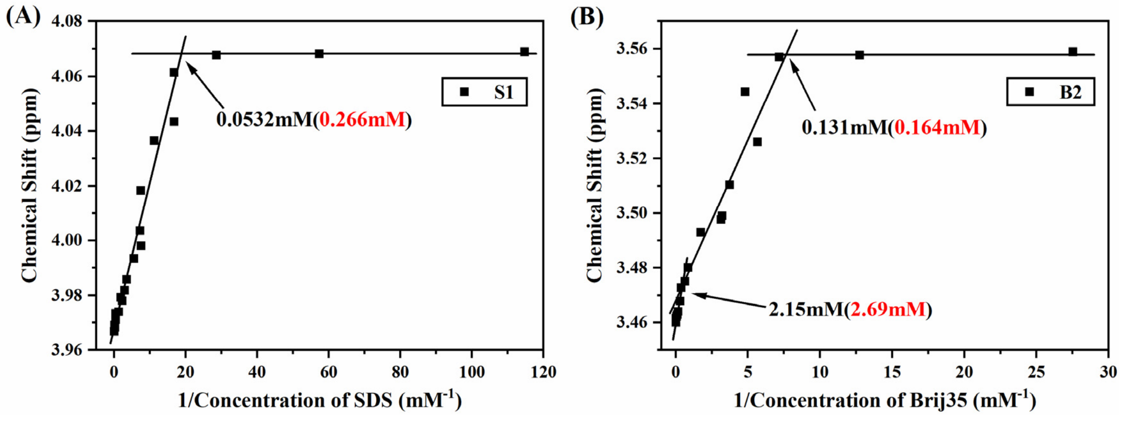

Figure S3 For α_{SDS}=0.2, variations of chemical shift of the resonance S1 from SDS (A) and B2 from Brij35 (B) as a function of reciprocals of their concentrations. The intersection of the fitted lines indicates the CMC, the corresponding C_T is shown in red font.

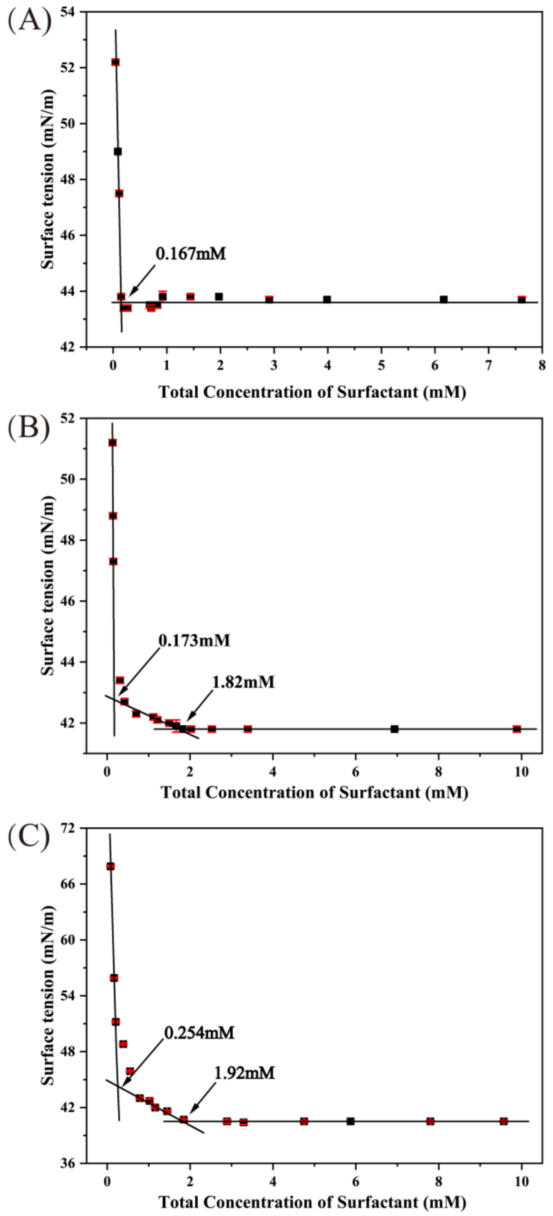

Figure S4 For α_{SDS}=0.2 (A), 0.5 (B) and 0.8 (C), variations in surface tension in SDS/Brij35 mixed systems solubilized by curcumin as a function of the total concentration of surfactant (C_T). The intersection of two lines indicates the CMC.

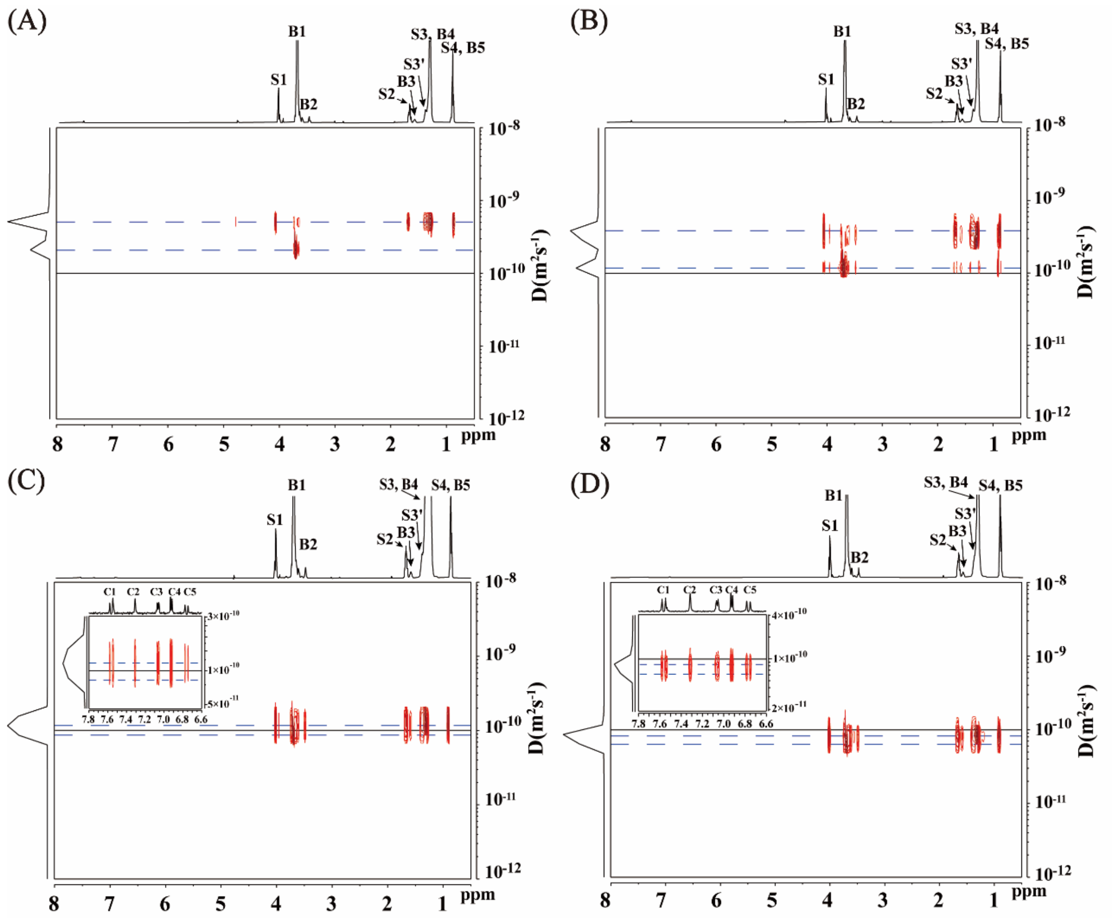

Figure S5 2D DOSY plots for the system with α_{SDS}=0.8 at C_T of 0.50 (A), 2.0 (B), 20 (C), and 40 mM (D). The black horizontal line at the D value of 10^{-10} m^2 s^{-1} is a reference to mark the variation of the diffusion coefficients.

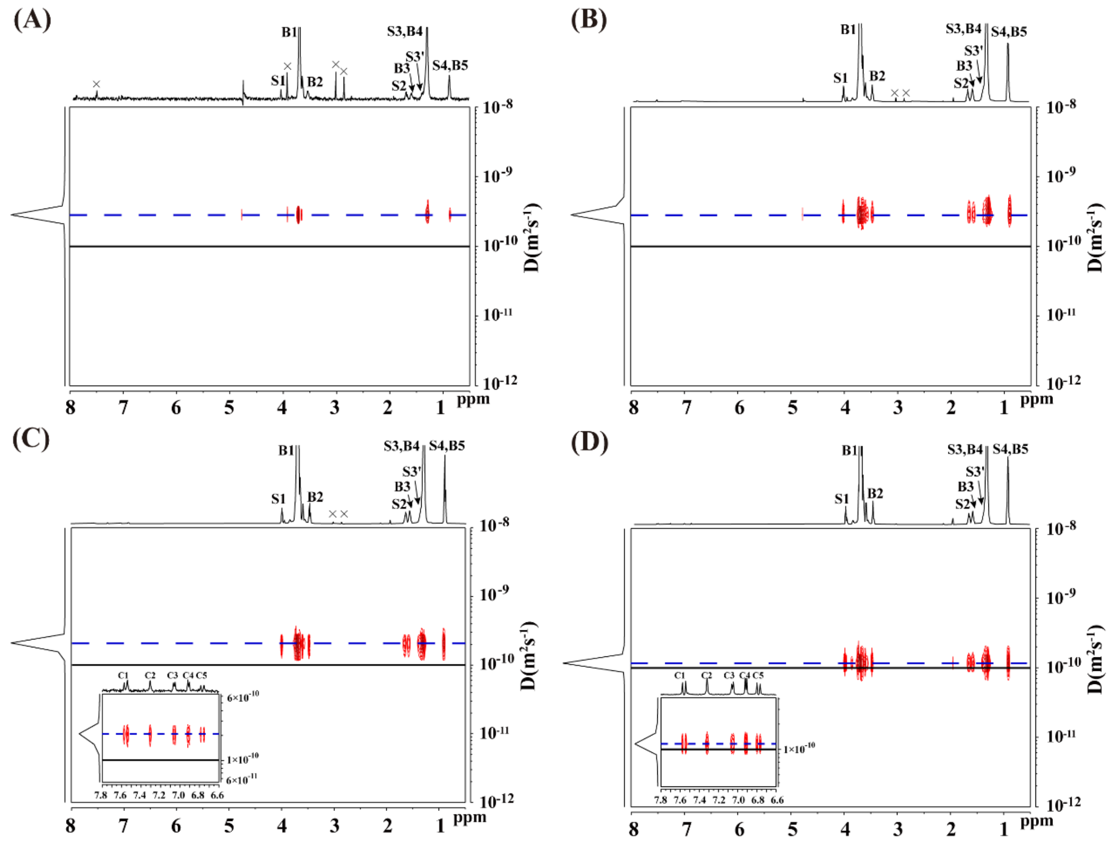

Figure S6 2D DOSY plots for α_{SDS}=0.5 at C_T of 0.20 (A), 2.0 (B), 8.0 (C), and 64 mM (D). The black horizontal line at the D value of 10^{-10} m^2 s^{-1} is a reference to mark the variation of the diffusion coefficients.

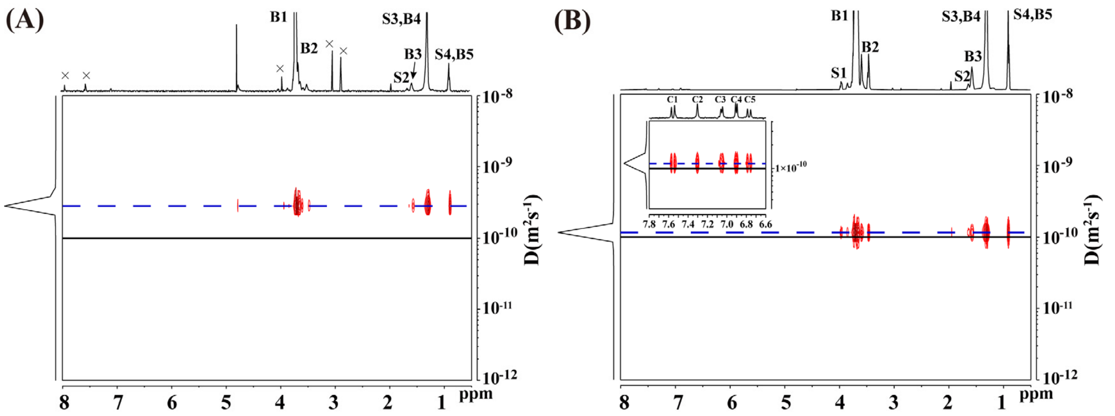

Figure S7 2D DOSY plots for α_{SDS}=0.2 at C_T of 0.20 (A) and 20 mM (B). The black horizontal line at the D value of 10^{-10} m^2 s^{-1} is a reference to mark the variation of the diffusion coefficients.

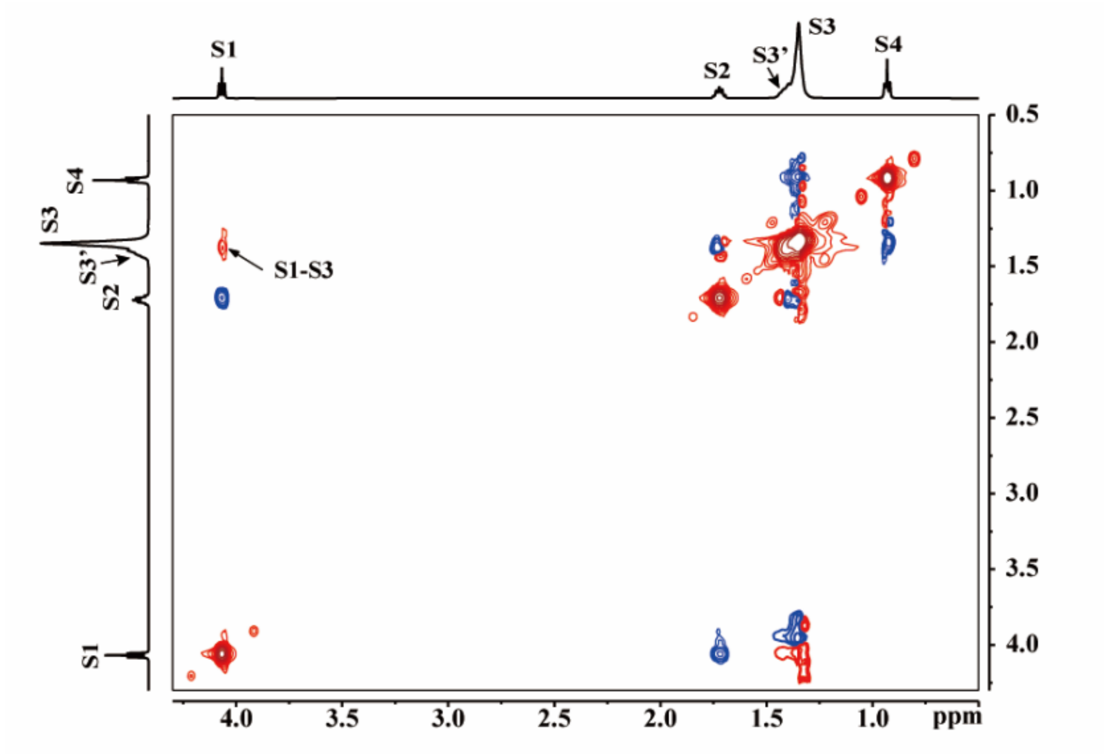

Figure S8 NOESY spectrum of the pure SDS solution at a concentration of 100 mM with a mixing time of 1 s.

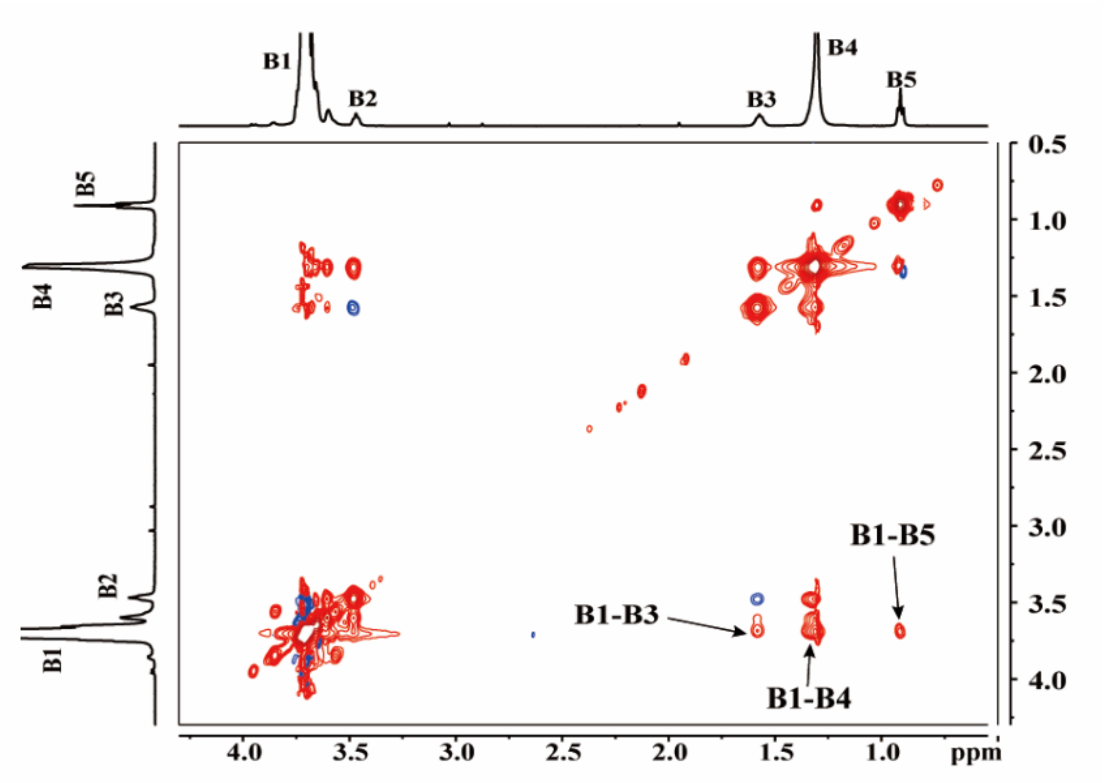

Figure S9 NOESY spectrum of the pure Brij35 solution at a concentration of 2.52 mM with a mixing time of 0.5 s.

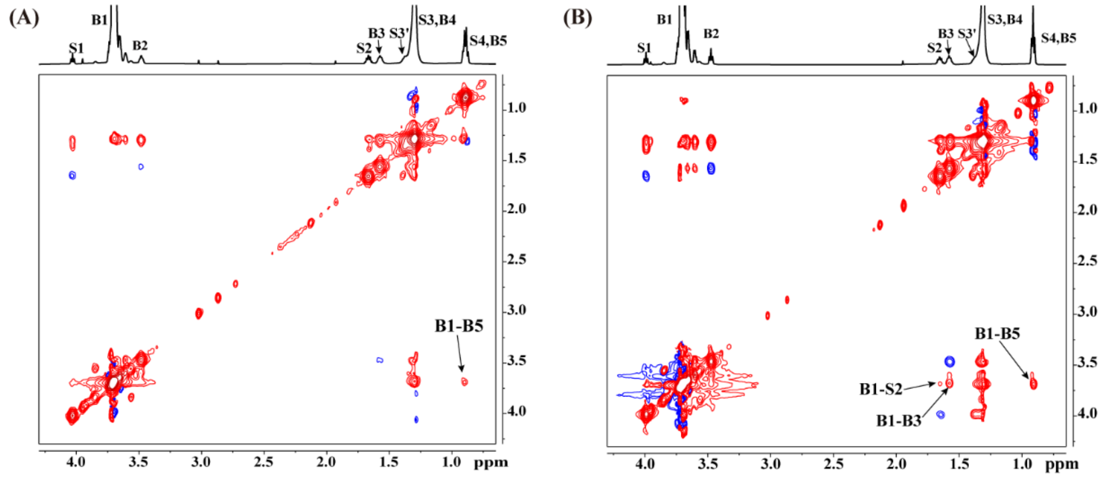

Figure S10 For α_{SDS}=0.5, NOESY spectra with a mixing time of 0.5 s at C_T of 1.0 (A) and 16 mM (B).

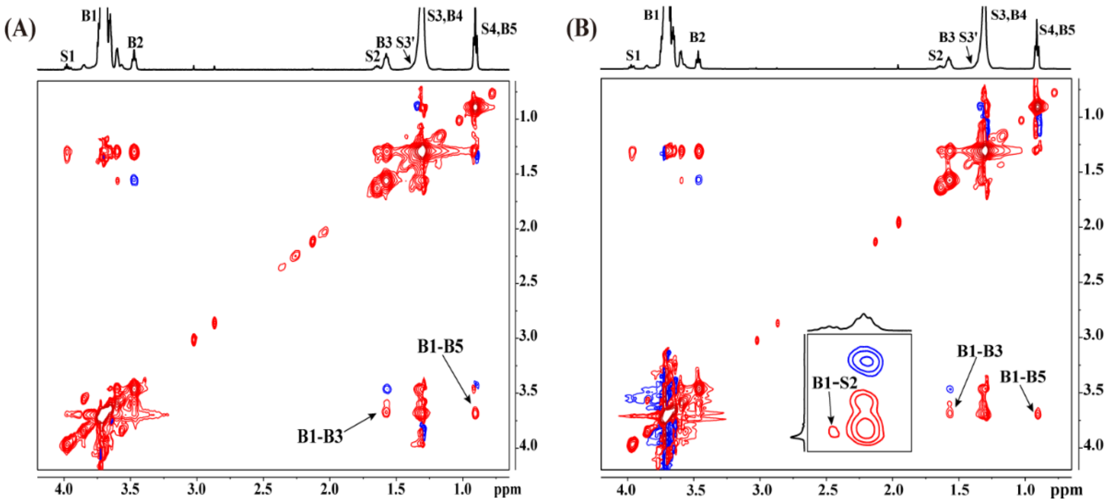

Figure S11 For α_{SDS}=0.2, NOESY spectra with a mixing time of 0.5 s at C_T of 2.0 (A) and 20 mM (B).

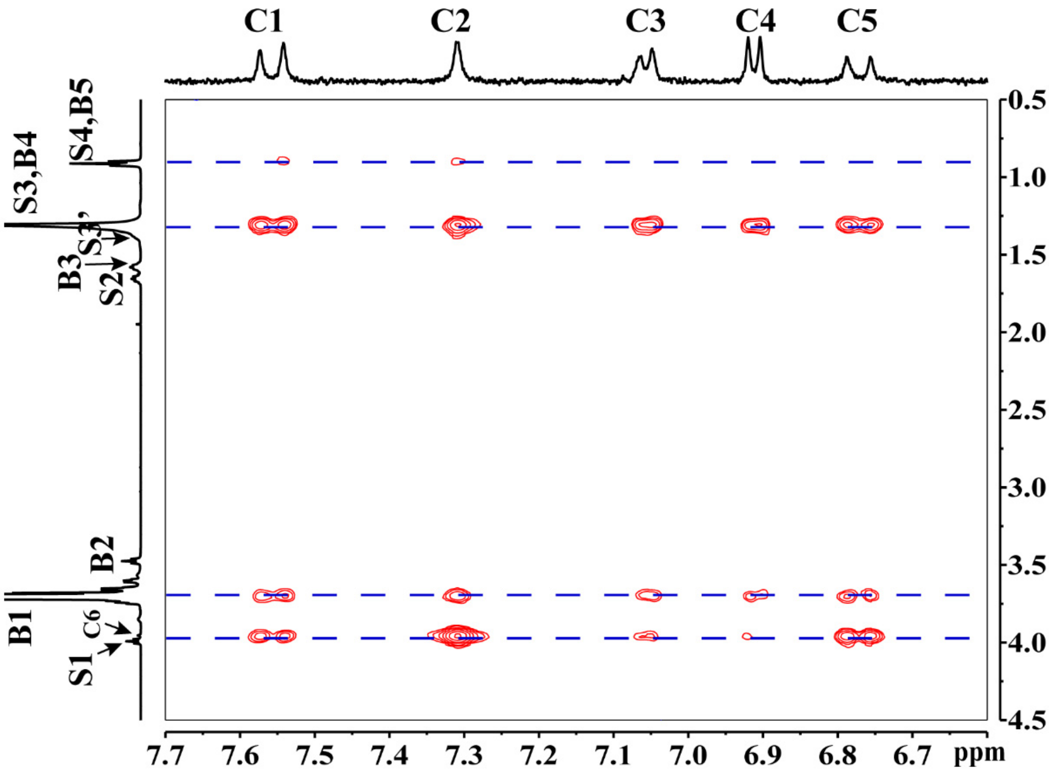

Figure S12 For α_{SDS}=0.5, at C_T of 16 mM, partially enlarged NOESY spectrum with a mixing time of 0.5 s.

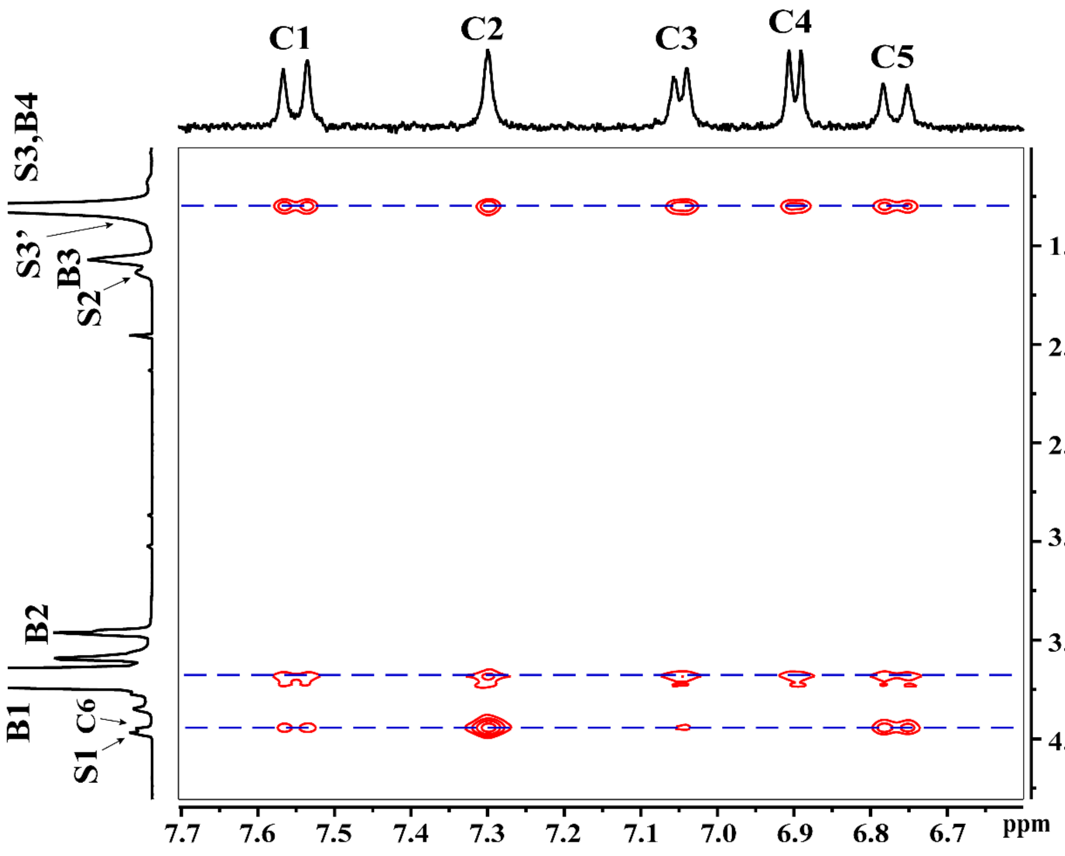

Figure S13. For α_{SDS}=0.2, at C_T of 20 mM, partially enlarged NOESY spectrum with a mixing time of 0.5 s.

Table S1. For α_{SDS}=0.8, hydrodynamic radii of kinetic units of SDS and Brij35 in aqueous solutions at varied concentrations, the solution viscosity is also shown.

C_T (mM)	Solution Viscosity (mPa. s)	Diffusion Coefficients ($10^{-10}m^2s^{-1}$)		Micelle Radius (nm)	
		SDS-rich micelles	Brij35-rich micelles	SDS-rich micelles	Brij35-rich micelles
**0.250**	0.99	5.13	2.12	0.430	1.04
0.500	0.99	5.13	2.12	0.430	1.04
2.00	1.00	3.81	1.17	0.573	1.87
5.00	1.03	2.12	0.64	1.00	3.31
10.0	1.04	2.42	1.59	0.868	1.32
20.0	1.10	1.34	0.862	1.46	2.30
40.0	1.11	0.862	0.640	2.28	3.08
80.0	1.15	0.640	0.640	2.97	2.97
